# Prognostic role of FDG PET/CT in patients with differentiated thyroid cancer treated with 131-iodine empiric therapy

**DOI:** 10.1097/MD.0000000000008344

**Published:** 2017-10-20

**Authors:** Barbara Salvatore, Michele Klain, Emanuele Nicolai, Domenico D’Amico, Gianluca De Matteis, Marco Raddi, Rosa Fonti, Teresa Pellegrino, Giovanni Storto, Alberto Cuocolo, Leonardo Pace

**Affiliations:** a Istituto di Biostrutture e Bioimmagini, CNR; b Dipartimento di Scienze Biomediche Avanzate, Università degli Studi di Napoli FedericoII; c IRCCS – SDN, Napoli; d Medicina Nucleare, IRCCS – CROB, Rionero in Vulture; e Dipartimento di Medicina, Chirurgia e Odontoiatria “Scuola Medica Salernitana,” Università degli Studi di Salerno, Salerno, Italy.

**Keywords:** ^18^F-FDG-PET/CT, differentiated thyroid carcinoma, empiric therapy, prognosis, thyroglobulin

## Abstract

**Background:**

To assess the long-term prognostic value of ^18^F-fluorodeoxyglucose (FDG) positron emission tomography/computed tomography (PET/CT) in patients with differentiated thyroid carcinoma (DTC) undergoing empiric radioiodine (RAI) therapy due to raising values of thyroglobulin (Tg).

**Methods:**

Forty-nine patients with histological diagnosis of DTC (31 with papillary and 18 with follicular carcinoma) follow-up for a mean period of 7.9 ± 5 years after empiric RAI therapy were retrospectively analyzed.

**Results:**

FDG-PET/CT was negative in 15 (30.6%) patients and positive in 34 (69.4%), whereas postradioiodine therapy whole body scan (t-WBS) was negative in 16 (32.7%) and positive in 33 (67.3%) patients. FDG-PET/CT and t-WBS were in agreement in 32 patients (7 both negative and 25 both positive); on the contrary, in 17 patients there was disagreement between FDG-PET/CT and t-WBS (*P* *=*ns). At short-term follow-up, Tg normalized in 19 (38.8%) patients and was unchanged or increased in 30 (61.2%). Of the 15 patients with negative FDG-PET/CT, 11 (73.3%) showed Tg normalization, whereas of the 34 patients with positive FDG-PET/CT, only 8 (23.5%) had Tg normalization (χ^2^ =8.9, *P* < .005). At multivariate analysis, FDG-PET/CT and Tg normalization at short-term follow-up were independent predictors of disease-free survival (χ^2^ =26.3, *P* < .0001), while Tg normalization was the only variable associated with overall survival χ^2^ =7.2, *P* < .01).

**Conclusion:**

FDG-PET/CT in association with Tg normalization at short-term follow-up may be useful for long-term prognostic stratification in DTC patients.

## 1 Introduction

Differentiated thyroid carcinoma (DTC) is usually characterized by a papillary and/or follicular pattern well represented on histologic examination and it has a very good prognosis, with an overall risk of relapse that never exceed 20%.^[1,2]^ Follow-up in patients with DTC consists of periodically measurements of serum thyroglobulin (Tg), Tg antibodies and neck ultrasound while diagnostic radioiodine whole body scan (d-WBS) is useful in patients with high or intermediate risk of persistent disease. Since the only cause of serum Tg is the presence of thyroid parenchymal cells, suggesting the presence of normal or metastatic thyroid tissue, generally patients with recurrence present elevated Tg levels and a new radioiodine (RAI) therapy may be performed. Serum Tg is recognized as the better marker to follow patients with DTC; however, the presence of anti-Tg antibody in the systemic circulation interferes with measurement of serum Tg.^[3]^^18^F-fluorodeoxyglucose (FDG) positron emission tomography/computed tomography (PET/CT) has became an important tool in the management of patients with DTC^[4]^ and in particular FDG-PET/CT may be useful in DTC patients with negative serum Tg but with persistently increased anti-Tg antibody level to point out metastases with higher malignant histological grade than the primary tumor.^[5]^ Moreover, a negative FDG-PET/CT, as we demonstrated in a previous study ,^[6]^ has a prognostic role in combination with serum Tg value at short-term follow-up in patients treated with empiric RAI therapy.

The most recent American Thyroid Association guidelines suggest RAI therapy in patients with stimulated serum Tg >10 ng/mL with thyroid hormone withdrawal or with Tg >5 ng/mL with recombinant human thyroid-stimulating hormone, rapidly rising serum Tg levels, or rising anti-Tg antibody levels, in whom imaging has failed to reveal a tumor source that is responsive to directed therapy.^[2]^ According to these guidelines, aspects to evaluate when selecting patients for empiric RAI therapy includes the level of serum Tg elevation and the results of FDG-PET/CT, if performed.^[2]^ Since thyroid cancers that show FDG uptake generally do not concentrate iodine-131 (^131^I),^[7]^ RAI therapy is much less likely to be efficacious^[2,6,7]^ and it is unlikely to alter the poorer outcome in such patients.^[8]^ Thus, it is recommended to perform FDG-PET/CT scanning prior to consider the administration of empiric RAI therapy.^[9]^ Metastatic or recurrent thyroid cancer positive at FDG-PET/CT often shows a higher malignant histological grade than the primary tumor, a pattern that is less common for DTC that are FDG-PET/CT negative.^[10]^ FDG-PET/CT has confirmed to be of prognostic value in DTC either in initial pretherapy evaluation^[11]^ or in posttherapy,^[12,13]^ as well as in metastatic patients.^[14,15]^ In a previous study, we demonstrated that at short-term follow-up after empiric therapy, the majority of patients with positive FDG-PET/CT scan still show elevated Tg levels while the opposite is true for patients with negative PET/CT.^[6]^ The aim of this study is to investigate the long-term prognostic role of FDG-PET/CT in patients with DTC undergoing empiric RAI therapy, extending the period of observation of our prior investigation.

## 2 Materials and methods

**Figure 1:**
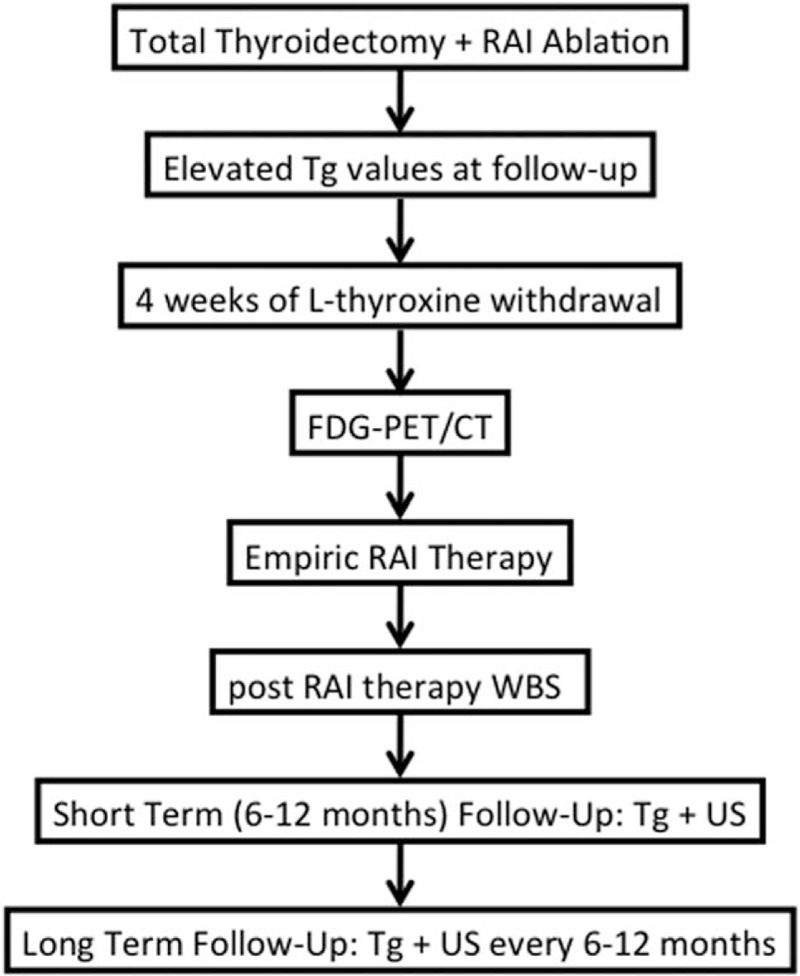
Management algorithm of DTC patients with elevated Tg values at long-term follow-up. DTC = differentiated thyroid carcinoma, Tg = thyroglobulin.

We reviewed retrospectively the medical records of 49 patients (29 women and 20 men; mean age 54.3 ± 17.4 years) with histological diagnosis of DTC (31 patients with papillary and 18 with follicular carcinoma) in part belonging to a previous series of patients studied by our group in the evaluation of short-term follow-up, that is, mean 15 ± 5 months.^[6]^ Briefly, all patients had persistently elevated Tg levels after total thyroidectomy and remnant ablation with RAI, no thyroid residual tissue and negative anti-Tg antibody. All patients received an empiric therapeutic dose of ^131^I (≥3700 MBq [megaBecquerel]) on the basis of elevated Tg values, after 4 weeks of L-thyroxine withdrawal and low iodine diet. FDG-PET/CT was performed 1 to 5 days before the empiric RAI therapy during thyroid-stimulating hormone levels ≥ 30 micro-International Units (μIU), while postradioiodine therapy whole body scan (t-WBS) was performed 5 to 6 days after RAI therapy (Fig. 1). The results of FDG-PET/CT did not have any impact on the decision to treat the patients.

Informed consent was obtained from all individual participants included in the study, as in our institution all patients sign an informed consent to the processing of data for scientific purposes. Ethical approval from the institutional review board was not necessary because this is a retrospective study.

### 2.1 Follow-up

All patients were followed up for a mean period of 7.9 ± 5 years (range 1–20 years). Objective outcome to therapy, that is, change in Tg and/or structurally evident disease by ultrasound, was assessed 6 to 12 months after RAI therapy and then yearly in each patient. All patients were followed until one of the clinical endpoints (recurrence, thyroid cancer-related death) was reached or censored on the last day of follow-up at our institution. A structurally progressive disease was defined as an increase more than 20% of any lesion or as an increase in the number of lesions foci; on the other hand a decrease in the size of 30% or a decrease in the number of lesions by at least 30% identified structural disease regression. Similarly, a 20% increase in suppressed Tg was defined as biochemical progression, and a 20% decrease was classified as biochemical regression.

### 2.2 Imaging studies

t-WBS and FDG-PET/CT images were performed in this series of patients as previously described.^[6]^ Briefly, t-WBS were acquired using a dual-head large field gamma camera with high-energy collimators (E.Cam, Siemens Medical Solutions). FDG-PET/CT scans were acquired before RAI therapy after fasting for 6 to 8 hours, 45 to 60 minutes after tracer administration (350–370 MBq) and glucose level, measured before tracer administration, was less than 140 milligram/deciliter (mg/dL), by using a combined PET/CT Discovery LS scanner (GE Healthcare Milwaukee).

All images were reviewed in consensus by 2 experienced nuclear medicine physicians (EN and DDA) that interpreted each set of images blinded to clinical, histopathological, and other imaging results. FDG-PET/CT and t-WBS were considered positive when at least 1 abnormal focus of tracer uptake was found, as previously described.^[6]^

### 2.3 Statistical analysis

All data are expressed as mean ± standard deviation or as percentage, as appropriate. Differences between groups were analyzed by Student *t* test or χ^2^ analysis, as appropriate. Univariate and multivariate analysis of variables were performed by Cox proportional hazards model. Only variables that predicted disease-free survival (DFS) and overall survival (OS) by univariate analysis were included in the multivariate analysis. Survival curves were generated using Kaplan–Meier analysis, and log-rank test for differences was used to assess significance. Survivors were censored at the time of last clinical control. A *P* value < .05 was considered to indicate a significant difference. MedCalc Statistical Software version 13.1.2 was used for statistical analysis (MedCalc Software bvba, Ostend, Belgium; http://www.medcalc.org; 2014).

## 3 Results

**Figure 2:**
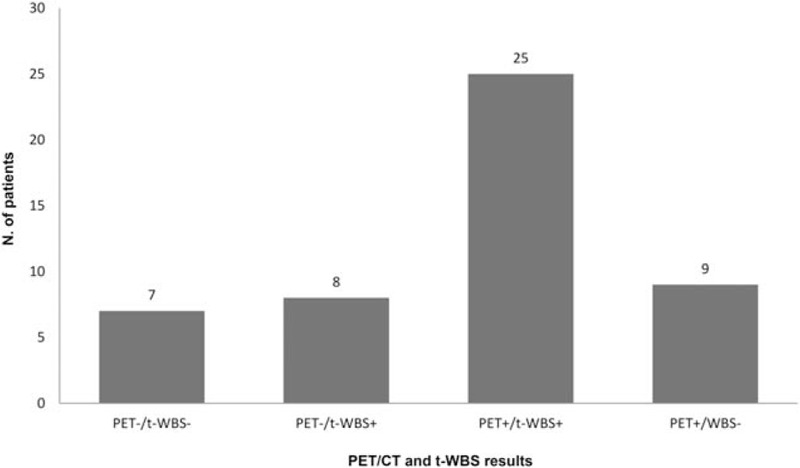
Comparison of ^18^F-FDG-PET/CT and t-WBS results in DTC patients. DTC = differentiated thyroid carcinoma, FDG-PET/CT = ^18^F-fluorodeoxyglucose-positron emission tomography/computed tomography, t-WBS = postradioiodine therapy whole body scan.

Clinical characteristics and FDG-PET/CT findings of the 49 patients studied are reported in Table 1. Of the 49 patients, 26 received 3700 MBq of ^131^I and 23 a higher activity (mean of the whole group: 4867 ± 1763 MBq). FDG-PET/CT performed immediately prior to empiric RAI therapy was negative in 15 (30.6%) and positive in 34 (69.4%) patients, showing loco-regional recurrence in 17 of them (50%) and distant metastases in the remaining group: bone in 5/17 (29.4%) and multiple sites in 12/17 (70.6%). t-WBS performed 5 to 7 days after empiric RAI therapy was negative in 16/49 (32.7%) and positive in 33/49 (67.3%) patients, showing loco-regional recurrence in 16/33 of them (48.5%) and distant metastases in the remaining 17/33: bone in 3/17 (17.6%) and multiple sites in 14/17 (82.4%). FDG-PET/CT and t-WBS were both negative in 7 patients and both positive in 25, while FDG PET/CT was negative and t-WBS was positive in 8 and the opposite was evident in 9 patients (*P* *=*ns) (Fig. 2). At short-term follow-up, Tg normalized in 19 patients (38.8%) and remained unchanged or increased in 30 patients (61.2%). At short-term follow-up only 4/15 (27%) patients with negative FDG-PET/CT showed Tg not normalized, while 26/34 (76%) patients with positive FDG-PET/CT presented Tg not normalized (χ^2^ = 8.9, *P* < .005).

**Table 1:**
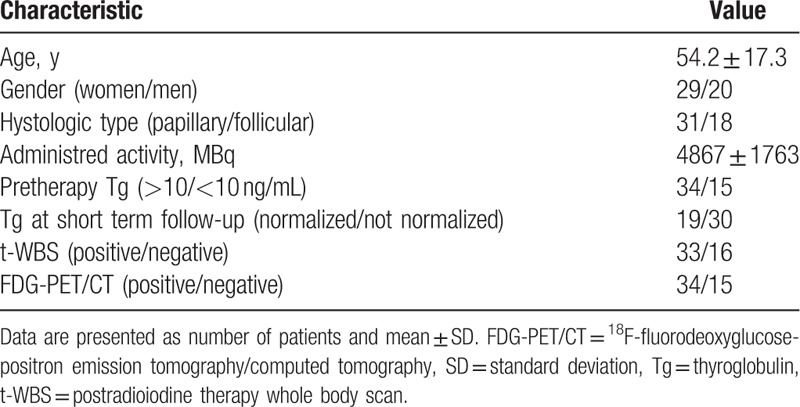
Patient characteristics.

**Figure 3:**
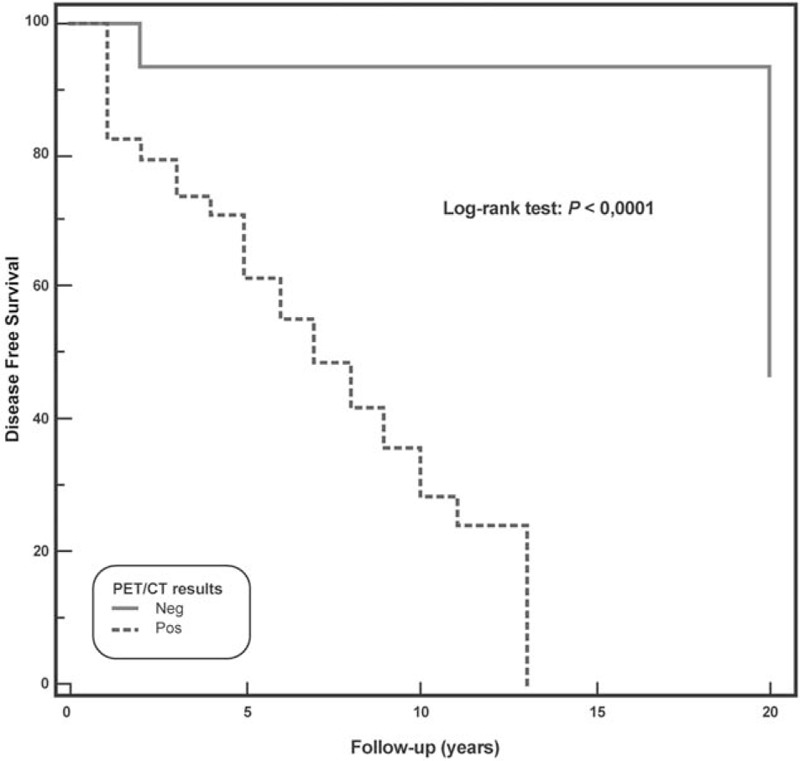
Disease-free survival by Kaplan–Meier analysis and log-rank test showing significant difference at 20 years between DTC patients with a positive ^18^F-FDG-PET/CT as compared to those with a negative ^18^F-FDG-PET/CT (*P* < .0001). DTC = differentiated thyroid carcinoma, FDG-PET/CT = ^18^F-fluorodeoxyglucose-positron emission tomography/computed tomography.

**Figure 4:**
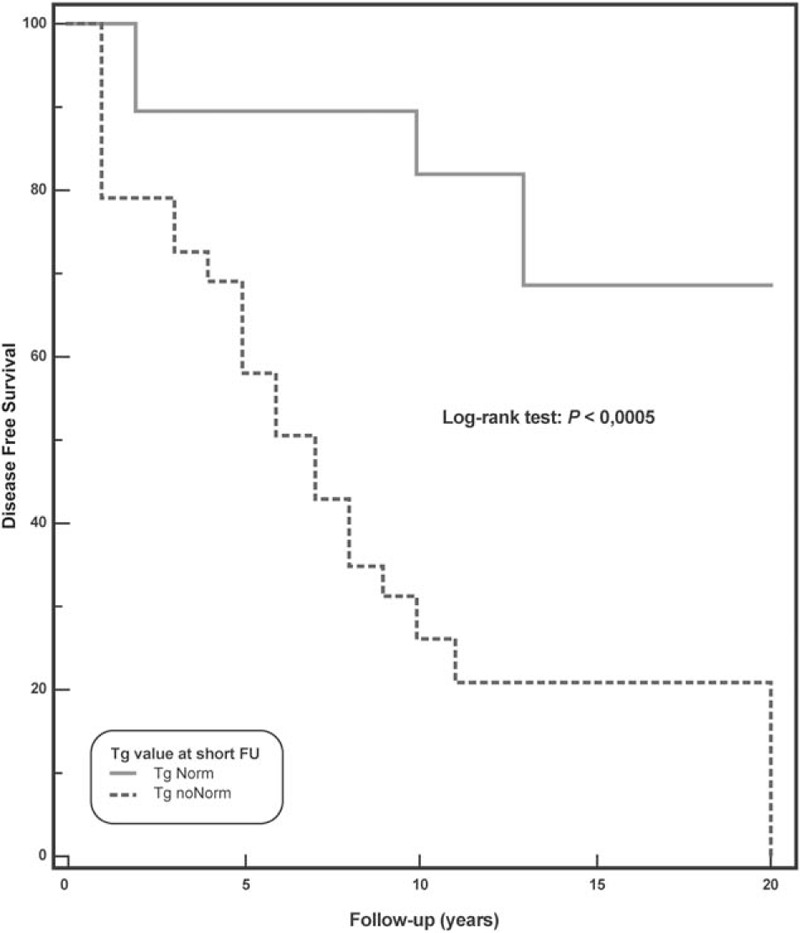
Disease-free survival by Kaplan–Meier analysis and log-rank test showing significant difference at 20 years between DTC patients with Tg normalization at short-term follow-up as compared to those without Tg normalization at short-term follow-up (*P* < .0005). DTC = differentiated thyroid carcinoma, Tg = thyroglobulin.

**Figure 5:**
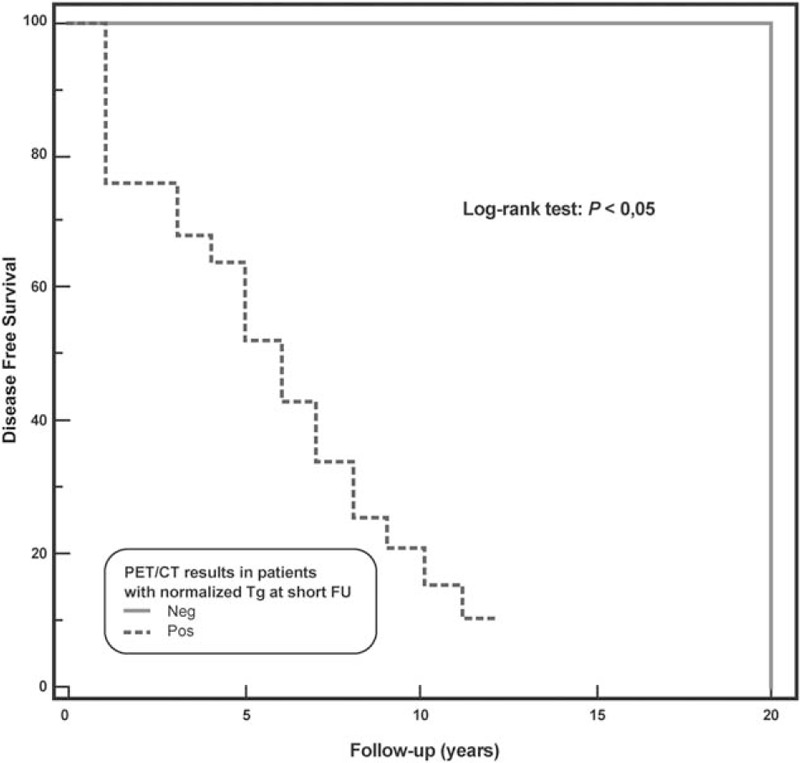
Disease-free survival by Kaplan–Meier analysis and log-rank test showing significant difference at 20 years between DTC patients, that showed Tg normalization at short-term follow-up, with a positive ^18^F-FDG-PET/CT as compared to those with a negative ^18^F-FDG-PET/CT (*P* < .05). DTC = differentiated thyroid carcinoma, FDG-PET/CT = ^18^F-fluorodeoxyglucose-positron emission tomography/computed tomography, Tg = thyroglobulin.

At long-term follow-up, relapsed disease was observed in 22 (44.9%) patients, while 5 died (10.2%). At univariate Cox analysis (Table 2), pretherapy Tg >10 ng/mL, administered activity >3700 MBq, Tg normalized at short-term follow-up, and FDG-PET/CT were significant predictors of DFS. At multivariate Cox model, Tg normalized at short-term follow-up and FDG PET/CT findings were the only independent predictors associated with DFS (χ^2^ = 26.3, *P* < .0001). Kaplan–Meier analysis showed a significant difference in DFS between patients with positive FDG-PET/CT scan and those with negative scan (log-rank, *P* < .0001, hazard ratio 0.19, 95% confidence interval 0.08–0.45) (Fig. 3) as well as between patients with Tg normalization at short-term follow-up and those without (long-rank, *P* < .0005, hazard ratio 0.22, 95% confidence interval 0.10–0.50) (Fig. 4). When only patients showing Tg normalization at short-term follow-up were considered, Kaplan–Meier analysis showed that FDG-PET/CT scan was a significant predictor of DFS (log-rank, *P* < .05, hazard ratio 0.24, 95% confidence interval 0.08–0.74) (Fig. 5).

**Table 2:**
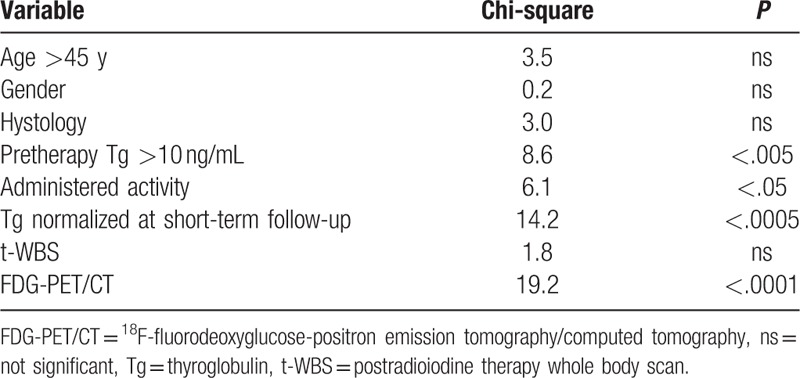
Predictors of disease-free survival at univariate analysis.

**Figure 6:**
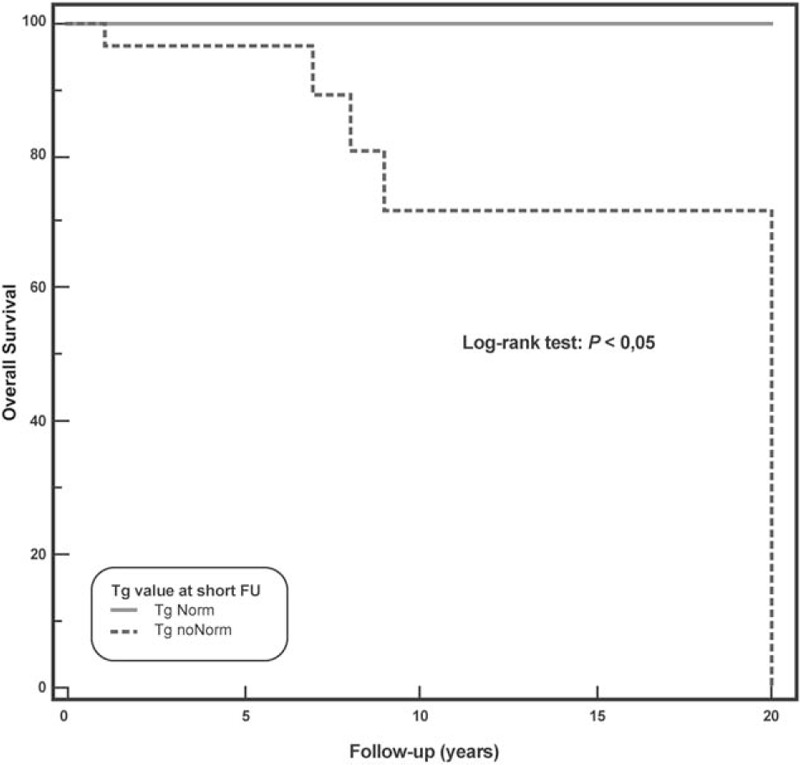
Overall survival by Kaplan–Meier analysis and log-rank test showing significant difference at 20 years between DTC patients with Tg normalization at short-term follow-up as compared to those without Tg normalization at short-term follow-up (*P* < .05). DTC = differentiated thyroid carcinoma, Tg = thyroglobulin.

Five (10.2%) of the 49 patients died. At univariate Cox analysis, age >45 years, pretherapy Tg >10 ng/mL, administered activity < 3700 MBq, Tg normalized at short-term follow-up, t-WBS, and FDG-PET/CT were significant predictors of OS (Table 3). At multivariate analysis, Tg normalized at short-term follow-up was the only variable associated with OS (χ^2^ = 7.2, *P* < .01). Kaplan–Meier analysis showed a significant difference in OS between patients with Tg normalization at short-term follow-up and those without (long-rank, *P* < .05, hazard ratio 0.12, 95% confidence interval 0.02–0.74) (Fig. 6).

**Table 3:**
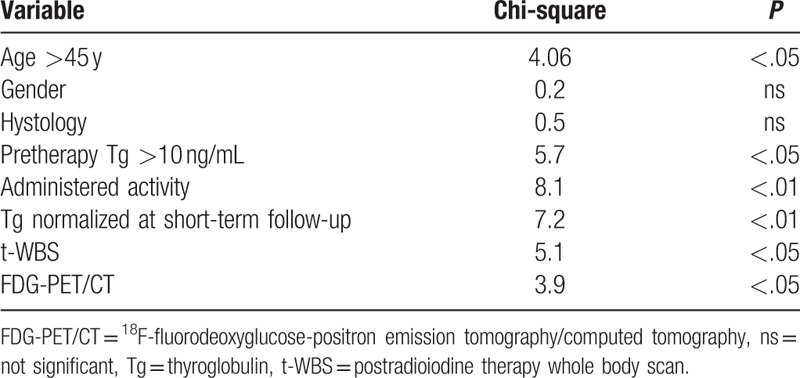
Predictors of overall survival at univariate analysis.

## 4 Discussion

The results of this study show that FDG-PET/CT is a useful tool to predict DFS in patients with DTC undergoing empiric RAI therapy, whereas Tg normalized at short-term follow-up was predictive of both DFS and OS. Moreover, FDG-PET/CT was able to predict DFS in patients with Tg normalization at short term follow-up.

The prognosis in DTC patients is usually excellent; however, it has been recognized that patients with distant metastases have a worse prognosis than those with loco-regional metastases^[16,17]^ as well as patients that have FDG avid lesions, elevated serum Tg postsurgery, and negative t-WBS or d-WBS.^[12]^ In this context, FDG-PET/CT improves the diagnostic accuracy, leading to a better tumor localization.^[18]^ Patients with DTC that have a low iodine uptake more frequently show higher uptake on FDG-PET/CT, as a marker of tumor dedifferentiation, in this subset of patients RAI therapy could be considered as an empiric therapy.^[9]^

In the last years, FDG-PET/CT has become the imaging technique of choice in oncologic patients^[19,20]^ becoming helpful in the decision-making process also in DTC patients in the evaluation both in the postoperative setting and in the restaging/follow-up recurrent thyroid carcinoma.^[6,11,14,21]^ The prognostic value of FDG-PET/CT in the management of DTC patients has been evaluated in many studies.^[6,12–15,22]^ Vural et al^[22]^ showed that a negative FDG-PET/CT predicts a benign prognosis, correlating with Tg levels at the follow-up. Similar results in a previous study by our group showed that FDG-PET/CT represents a prognostic tool combined with serum Tg at short-term follow-up in DTC patients undergoing empiric RAI therapy due to raising values of Tg.^[6]^ In the present study, we demonstrated that in patients, undergoing empiric RAI therapy because of elevated Tg values, FDG-PET/CT was a strong predictor of DFS at long-term follow-up both alone and in association with Tg normalized at short-term follow-up. Actually, patients with negative FDG-PET/CT had a 10 years DFS of 93% versus 28% of those with positive FDG-PET/CT (*P* < .0001), while 10-years DFS was 82% among patients with Tg normalized at short-term follow-up versus 26% in those without (*P* < .0005). It should be noted that in the subgroup of patients with Tg normalized at short-term follow-up, FDG-PET/CT was a powerful predictor: patients with a negative FDG-PET/CT had a 10-years DFS of 100% while those with a positive FDG-PET/CT showed a 10-years DFS of 16% (*P* < .05).

It is well known that FDG-PET/CT is a useful tool to individuate metastases with higher malignant histological grade,^[5]^ and thus in our study a positive FDG-PET/CT could represent an indicator of increased aggressiveness predicting relapses. On the other hand, FDG-PET/CT is able to exclude recurrence or metastases^[24]^ and negative FDG-PET/CT could be predictive of a very good response to therapy.^[25]^ Similar to our findings, other retrospective studies demonstrated that a negative FDG-PET would predict an excellent outcome despite an elevated Tg value.^[13,14]^ Likewise a study on the outcome of medical and surgical management in DTC patients showed that a positive FDG-PET predicts a worse prognosis and can help to distinguish between patients with distant metastases and not candidates for reoperation and patients with localized neck lesions that should be resected.^[23]^

It has been reported that FDG-PET/CT is also predictive of OS in DTC patients with high serum Tg or with advanced stage cancers,^[13,24]^ while in our study only Tg normalized at short-term follow-up was predictive of OS: the 9-years OS was 100% in patients with Tg normalized at short-term follow-up and 72% in those without (*P* < .05). The lack of predictive value of FDG-PET/CT for OS in the our results could be due to the relatively small of number of patients, which should be acknowledged as one of the limitations of our study together with its retrospective nature.

The findings of this study show that in DTC patients FDG-PET/CT is useful in the follow-up of those with high Tg levels to localize metastatic lesions and to establish appropriate treatment^[6,13]^ and FDG-PET/CT, alone or in combination with Tg, provides prognostic information able to predict any relapses. Moreover, our results suggest that FDG-PET/CT could be used to select those patients in whom RAI empiric therapy may be more effective.
